# Point-of-Care Diagnosis of Atrophic Gastritis by Serological Biomarker Test (GastroPanel^®^ Quick Test) in Gastroscopy Referral Patients in India

**DOI:** 10.3390/jcm14030787

**Published:** 2025-01-25

**Authors:** Mohinish Chhabra, Ajit Kolatkar, Suresh Chawla, Aniket Joshi, Marika Karjalainen, Heli Holopainen, Panu Hendolin, Kari Syrjänen

**Affiliations:** 1 GI Physiology and Motility Laboratory, Department of Gastroenterology, Fortis Hospital and Research Centre, Sector 62, Lamba, Sahibzada Ajit Singh Nagar 160062, Punjab, India; chhabramohinish@gmail.com (M.C.); suresh_2905@yahoo.co.in (S.C.); 2 GastroLab India Pvt Ltd., 202, Specialy Business Centre, Balewadi Rd, Balewadi, Pune 411045, Maharashtra, India; dr.ajit@gastrolab.com (A.K.); aniket.joshi@gastrolab.com (A.J.); 3 Department of Clinical Research, Biohit Oyj, 00880 Helsinki, Finland; marika.karjalainen@biohit.fi (M.K.); heli.holopainen@biohit.fi (H.H.); panu.hendolin@biohit.fi (P.H.); 4 SMW Consultants, Ltd., Kylliäisentie 9, 21620 Kaarina, Finland; 5 Molecular Oncology Research Center, Barretos Cancer Hospital, Barretos CEP 14784-400, Brazil

**Keywords:** atrophic gastritis (AG), serological biomarker panel, GastroPanel^®^ Quick Test, non-invasive diagnosis, clinical validation, diagnostic accuracy (DA), gastroscopy, biopsies, Updated Sydney System (USS), *Helicobacter pylori*, pepsinogen I, pepsinogen II, gastrin-17, *Hp* IgG antibody

## Abstract

**Background:** Increased demand of the serological biomarker test (GastroPanel^®^) in non-invasive diagnosis of gastric cancer (GC) risk conditions, i.e., atrophic gastritis (AG) and *Helicobacter pylori* (*Hp*) infection, prompted the design of GastroPanel^®^ Quick test (GPQT) (Biohit Oyj, Helsinki, Finland) for point-of-care (POC) settings. **Objective:** This study validated the diagnostic accuracy (DA) of GPQT in diagnosis of AG and *Hp* among gastroscopy referral patients. **Methods:** Altogether, 266 patients were enrolled among the consecutive gastroscopy referrals at the Department of Gastroenterology, Fortis Hospital (Punjab, India). All patients underwent gastroscopy with biopsies (n = 249) classified using the Updated Sydney System (USS) and finger prick blood sampling for GPQT testing. **Results:** Biopsy-confirmed AG was found in 15.3% (38/249) of the patients. The overall agreement between the GPQT and the USS classification was 71.4% (95% CI 65.4–77.0%), with the weighted kappa (κ_w_) of 0.823 (95% CI 0.773–0.862). In ROC analysis for moderate/severe AG of the corpus (AGC) endpoint, AUC = 0.990 (95% CI 0.979–1.000) and AUC = 0.971 (95% CI 0.948–0.995) for PGI and PGI/PGII, respectively. *Hp* IgG Ab test detected biopsy-confirmed *Hp* with AUC = 0.836 (95% CI 0.783–0.889). **Conclusions**: The GPQT favourably competes in accuracy with the ELISA test version (unified-GP) in diagnosis of AG and *Hp* in patients referred for diagnostic gastroscopy.

## 1. Introduction

In India, gastric cancer (GC) is the seventh most common cancer, with annual incidence of 4.5 per 100,000 population (64,600 new cases) and annual mortality of 4.1 per 100,000 population (57,000 GC deaths) [1]. Two risk factors for GC exceed all the others in importance: (i) *Helicobacter pylori* (*Hp*) infection and (ii) atrophic gastritis (AG) [2,3,4]. *Hp* is a Group 1 human carcinogen (IARC), known to induce GC via virulence factors, such as cagA, by interfering with intracellular signaling and promoting neoplastic transformation of gastric epithelial cells [5]. *Hp*-induced AG is the single most important high-risk condition of GC [2,4,6]. The risk of GC increases with the severity of AG; a 90-fold increase is seen in patients with severe AG both in the corpus and in the antrum, i.e., pan-gastritis (AGP) [2,3,4,7]. Of all *Hp*-infected patients (40–50% of the world population), 5–10% are estimated to develop moderate to severe AG if *Hp* is not effectively eradicated [7].

GC develops through Correa cascade [2], with mild, moderate and severe AG as intermediate steps [2,4]. It is not clear whether this cascade can be interrupted or reversed through the eradication of *Hp*-infection [3,6,8]. According to the Updated Sydney System (USS) classification, AG is classified by its topographic location in the body (antrum, corpus, or both) as AGA, AGC, or AGP, respectively [9]. AG with or without *Hp*-involvement is frequently asymptomatic but is also a known cause of upper abdominal symptoms (dyspepsia) [9,10].

AG is usually diagnosed by esophago-gastro-duodenoscopy (EGD) and biopsy [2,9,11]. As an invasive method, EGD is expensive, not without complications, and causes discomfort in many patients. There is thus a need for non-invasive tests in diagnosis of AG [12]. The early studies in Japan testing pepsinogens (PGs) as serological biomarkers of AG [13,14] paved the way to the design of a 4-biomarker panel (GastroPanel^®^ test) that combines serum pepsinogen I (PGI) and II (PGII), gastrin-17 (G-17), and Hp antibody (Hp IgG) as an ELISA test, by Biohit Oyj (Helsinki, Finland) [15]. Primarily used in diagnosis and screening of *Hp*-infections and AG, the GP test also measures the state of gastric acid output. However, it does not cover all potential causes of functional dyspepsia [12,15,16]. GastroPanel^®^ is a tool for (i) non-invasive diagnosis of dyspeptic patients for exclusion or confirmation of AG, also measuring (by G-17) the status of gastric acid output; (ii) screening of asymptomatic individuals at risk of GC; and (iii) comprehensive diagnosis of *Hp*-infections. By integrating the biomarker profiles with the individual patient’s medical information, GastroPanel^®^ test is capable of distinguishing eight diagnostic categories (five profiles of gastric mucosal structure and three profiles of stomach function) [16].

The GP test has gained increasing global interest, with a rapidly increasing number of diagnostic accuracy (DA) studies conducted in both clinical and screening settings [17,18,19,20,21,22,23,24,25,26,27,28,29]. Accumulated literature has been covered by three separate meta-analyses [30,31,32], consistently reporting pooled sensitivity (SE) of 70–75% and pooled specificity (SP) close to 95% for the GP test in diagnosing AGC. The latest ELISA version of the GP test (unified-GP) harmonizes the laboratory processing conditions of the four biomarkers [33,34]. GastroPanel^®^ quick test (GPQT) was developed for point-of care (POC) diagnosis during a single clinical appointment [35]. The results of GPQT (a lateral-flow test) are read in 15 minutes with the GastroPanel^®^ Reader instrument (GP reader NT) [36], and their interpretation is based on the diagnostic GastroSoft^®^ algorithm [16,25,33,34,35].

This is the first clinical validation study of GPQT in a cohort of gastroscopy referral patients in a major hospital in India (Fortis Hospital, Sahibzada Ajit Singh Nagar, Punjab, India). The endoscopy capacity in this country is overloaded, which offers a rationale for adopting GPQT: to (i) obtain cost savings by reducing unnecessary clinical appointments, (ii) speed up the access to clinical triage (EGD) of the patients whenever indicated, and (iii) allow more rational allocation of the endoscopy capacity while unnecessary EGD examinations can be avoided [35].

## 2. Materials and Methods

### 2.1. Materials

The patients were enrolled at the outpatient Department of Gastroenterology, Fortis Hospital, Mohali Chandigarh, India, among the consecutive patients referred for gastroscopy due to upper abdominal symptoms (dyspepsia). The prospective study participants (aged 18 years or older) were identified among the gastroscopy referral outpatients, and they were asked to sign a written consent. The exclusion criteria were the same as listed in the recent validation studies of other GP test versions [26,27,33]. The following patients were considered ineligible to participate: (1) those whose treatment requires surgery, or immediate follow-up treatment for major symptoms, and (2) those who refused to participate. The study protocol followed the Declaration of Helsinki and was approved by the Institutional Ethics Committee (Fortis Hospital) (GPQT-UGIE-1; 2 September 2020).

A cohort of 266 patients completed the study protocol. The key characteristics of the patients and their history of dyspepsia-related symptoms are summarized in Table 1. Of the 266 patients, 50.8% were women and 49.2% were men. The median age of the patients was 52 years (their age range spanned 18 to 92 years).

### 2.2. Methods

#### 2.2.1. Preparation for GPQT Sampling

Instructions on the pre-sampling preparatory measures were given to the patient at the time of his/her consent to participate. The patient was instructed to follow a 10-hour fasting overnight [34,35]. They were encouraged to discontinue using gastric acid blockers (PPI-medication and H_2_-receptor antagonists) seven days before the date of GPQT sampling [35], while medication neutralizing gastric acid secretion (i.e., antacids and mucosa protecting agents) was allowed to continue until the preceding day [15,16].

#### 2.2.2. Completion of the GPQT Referral Form

A complete record of the Referral Form is an essential part of GPQT testing, because the GastroSoft^®^ application incorporates the key clinical information with the biomarker profile to create the final GPQT report [15,16,34]. The clinical information recorded in the referral form includes the following specific questions: (1) Do you have a history of previously eradicated Hp-infection? If yes, was this done less or over a year ago? (2) Do you use gastric acid blockers, and if so, what is the interval (in days) in their use before GPQT sampling? (3) Do you have reflux symptoms due to high acid output? and (4) Do you use of non-steroidal anti-inflammatory drugs (NSAIDs)? [26,27,34,35].

#### 2.2.3. GastroPanel^®^ Quick Test (GPQT)

GPQT is a lateral-flow test, based on immunoassay method detecting four biomarkers, namely PGI, PGII, G-17, and HpAb from a blood sample (whole blood, plasma, or serum) [35]. The capability of the GPQT to provide the test results during a single clinical visit is a major advantage to the GastroPanel^®^ ELISA versions analyzed in a clinical laboratory [15,16,34].

#### 2.2.4. Sample Collection for GPQT

For GPQT, the whole blood sample can be obtained from fingertip blood drop. Finger-prick blood samples (2 × 40 µL) are collected with disposable micropipettes and transferred into the sample tubes pre-filled with sample dilution buffer [35].

#### 2.2.5. Sample Processing for GPQT

Dispensed samples are transferred with disposable transfer pipettes from the sample tubes into the four sample holes (100 µL into each hole) of the lateral-flow test cassette. The cassette is measured after 15 minutes using the GastroPanel^®^ Reader instrument (GP reader NT) [36]. Results can be read on the reader screen or printed. The printed report shows both numerical values of all biomarkers and a written interpretation of the test result [15,16,26,27,33,34,35].

#### 2.2.6. Interpreting the GPQT Results

The results of the GPQT are interpreted by GastroSoft^®^ application, designed for use with the Updated Sydney System (USS) classification of gastritis [9,11]. Both use the same diagnostic categories: (1) normal stomach mucosa, (2) Hp-induced gastritis (with no atrophy), (3) atrophic antrum gastritis (AGA), (4) atrophic corpus gastritis (AGC), and (5) atrophic pan-gastritis (AGP) [9,11,16,26,27,33,35,36].

#### 2.2.7. Gastroscopy and Biopsies

Esophago-gastro-duodenoscopy (EGD) was performed by certificated endoscopists at the Department of Gastroenterology, Fortis Hospital, using videoscopes (GIF-H240, GIF-H260, or GIF-HQ290, Olympus, Tokyo, Japan) and following the routine procedures. On EGD, the gastric mucosal atrophy was evaluated according to the endoscopic atrophic border scale described by Kimura and Takemoto (K-T classification) [37]. K-T is an endoscopic classification of gastric atrophy as either (1) closed (C) or (2) open (O) type, both being graded from 0 to III (C-0 to C-III, and O-0 to O-III) [37], where Grade 0 denotes no atrophy. While performing EGD, the endoscopists were blinded to the GPQT results. Apart from the K-T classification, a detailed description of all observed mucosal abnormalities (including impression of atrophy) were given, including their location in the esophagus, stomach, duodenal bulb, and the second part of the duodenum.

#### 2.2.8. Biopsy Protocols

Directed biopsies were taken according to the sampling protocol of the Updated Sydney System (USS). In each patient, at least two routine biopsy specimens were taken from each area, the antrum and corpus [9,11]. In addition, two extra biopsies were taken from the incisura angularis, as well as from the bulb, the second part of the duodenum, distal and mid esophagus [9,11]. Endoscopic biopsies were used as the reference (gold standard) test in calculating the DA of GPQT.

#### 2.2.9. Interpretation of the Gastric Biopsies

All biopsies were examined by expert pathologists at the Department of Pathology, Fortis Hospital, and classified using the USS classification [9,11] as normal, Hp-gastritis, AGA, AGC and AGP (called USS original). All biopsies originally classified as AG (AGA, AGC or AGP) at Fortis Hospital were subjected to whole slide scanning by OS Ultra Scanner (OptraSCAN India Private Ltd., Pune, Maharashtra, India). The scanned whole slides were made available for reviewing (in Finland) with OS Image Viewer (Optra IMAGEPath). The slides reviewed in Finland (by KS) were also classified using the USS system (named USS Revised).

#### 2.2.10. Statistical Analyses

Sensitivity (SE), specificity (SP), positive predictive value (PPV), negative predictive value (NPV), and their 95% CI of the GPQT biomarkers were calculated using the algorithm of Seed et al. (2001) [38]. ROC (Receiver Operating Characteristics) analysis was used to identify the optimal SE/SP balance for AGA and AGC endpoints. AUC values were compared by the Roccomb test [26,27]. The agreement between the different classifications was calculated using overall agreement (OA) and intra-class correlation coefficient (ICC) test for weighted kappa (κ_w_). In addition, Fagan’s nomogram [39] was constructed to give the post-test predictions for AGC at the population level, based on the calculated indicators: (i) the pre-test probability; (ii) positive likelihood ratio (LR+), and (iii) negative likelihood ratio [39]. Statistical analyses were performed using either SPSS 29.0.2.0 for Windows (IBM, Armonk, NY, USA) or STATA/SE 18.0 software (STATA Corp., College Station, TX, USA). All tests were interpreted as significant at the level of *p* < 0.05.

## 3. Results

Table 1 (above) summarizes the patients’ medical history recorded in the GPQT referral forms. Both male and female patients were equally represented, and the median age of the patients was 52.0 years (with a range of 18 to 92 years). *Hp*-infection was not previously diagnosed in the vast majority (83.8%) of the patients, and *Hp*-eradication was given to 24 of 266 (9%) patients only. Regular use of PPI medication was reported by 74% of the patients, and regular symptoms due to high acidity were reported by 97%. The possible discontinuation of PPI-medication before GPQT sampling was not accurately recorded, and the same was true with the history of NSAID (non-steroidal anti-inflammatory drugs) medication.

Table 2 depicts the biomarker values (M ± SD) in the five diagnostic categories of the GPQT. The values of all biomarkers fall within the range of the manufacturer’s reference values in each diagnostic category, except for G-17b, which shows values exceeding the reference range in two categories: normal, Hp-gastritis. In these two categories, the PGI values are also close to the upper limit (160 μg/L).

Table 3 lists the biomarker levels stratified by the gastric biopsy histology (revised USS classification). The biomarker values in the five categories of the USS classification closely follow those of the five GPQT categories. The levels of G-17b values are substantially higher in the USS AGA category as compared with those in the GPQT AGA.

The agreement between GPQT and USS classification is shown in Table 4. Based on the revised USS, the stomach was graded normal in 146 of 249 (58.6%) of the patients. *Hp*-associated superficial gastritis (with no AG) was found in 65 of 249 (26.1%) of the patients, AGA was diagnosed in 21 patients, AGC in 15 cases and AGP in two patients (Table 4). The OA between the GPQT and the USS classification is 0.714 (i.e., 71.4%). The GPQT-USS agreement has κ_w_ = 0.823 (95% CI 0.773–0.862).

The DA of the (GastroSoft^®^) AGA- and AGC-profiles in detection of biopsy-confirmed AGA and AGC is summarized in Table 5. For AGA and AGA2+ endpoints, SE and PPV of the GPQT AGA profile are poor, while SP and NPV are high, resulting in AUC values between 0.569 and 0.648. In contrast, the AGC-profile predicts biopsy-confirmed AGC and AGC2+ with high accuracy, AUC = 0.933 and AUC = 0.977, respectively.

Table 6 summarizes the diagnostic accuracy of GPQT biomarkers (PGI, PGI/PGII) and G-17b in detection of AGC and AGA, respectively, using two different cut-offs for PGI. G-17b is of limited value in diagnosing AGA and AGA2+, with AUC values of 0.532 and 0.585, respectively. Using the AGC2+ endpoint, the best DA (AUC = 0.983) is obtained for PGI (30 μg/L), followed by PGI (15 μg/L) (AUC = 0.929). The PGI/PGII ratio (cutoff 3.0) did not reach the DA of PGI for either AGC or AGC2+.

**Figure 1:**
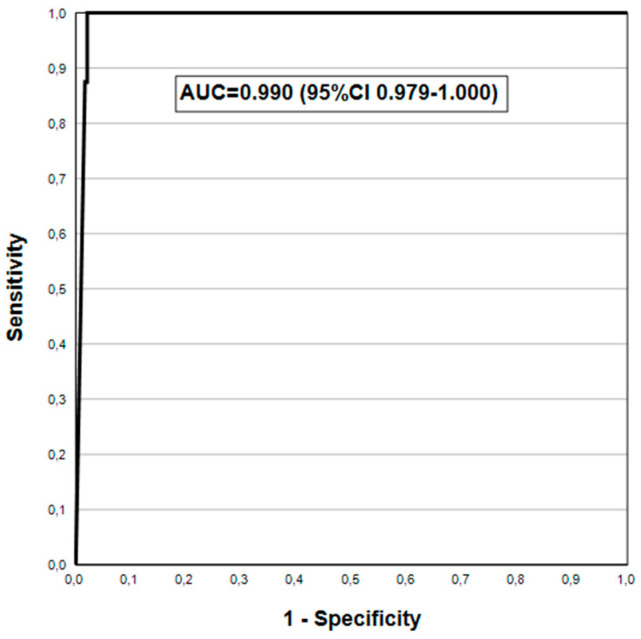
PGI in detecting biopsy-confirmed moderate/severe AGC in ROC analysis.

Figure 1 shows the ROC curve for PGI using the AGC2+ endpoint, with the AUC = 0.990 (95% CI 0.979–1.000).

**Figure 2:**
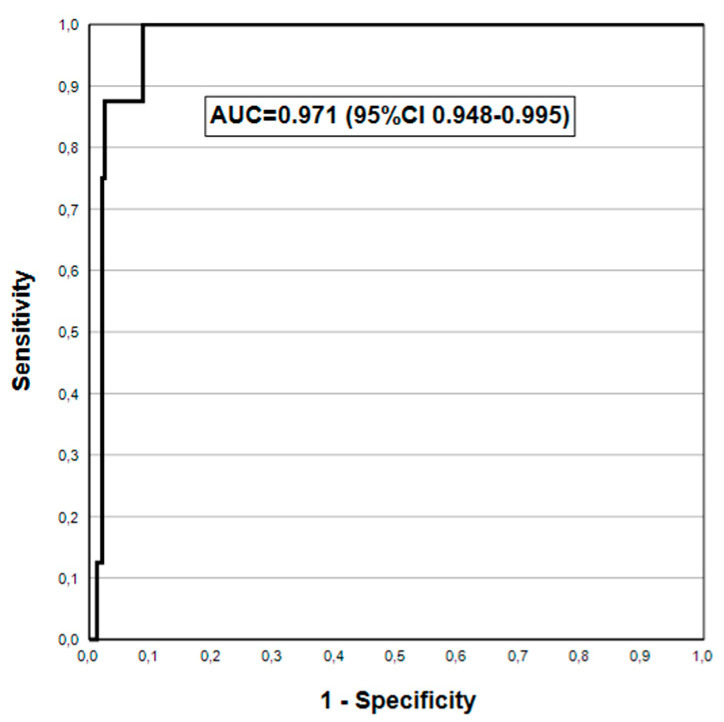
PGI/PGII ratio in detecting biopsy-confirmed moderate/severe AGC in ROC analysis.

The ROC curve for PGI/PGII ratio is only slightly inferior, with the AUC = 0.971 (95% CI 0.948–0.995) (Figure 2). The DA of fasting G-17 (G-17b) in detection of AGA2+ was clearly inferior in ROC analysis, with AUC = 0.675 (95% CI 0.480–0.870).

**Figure 3:**
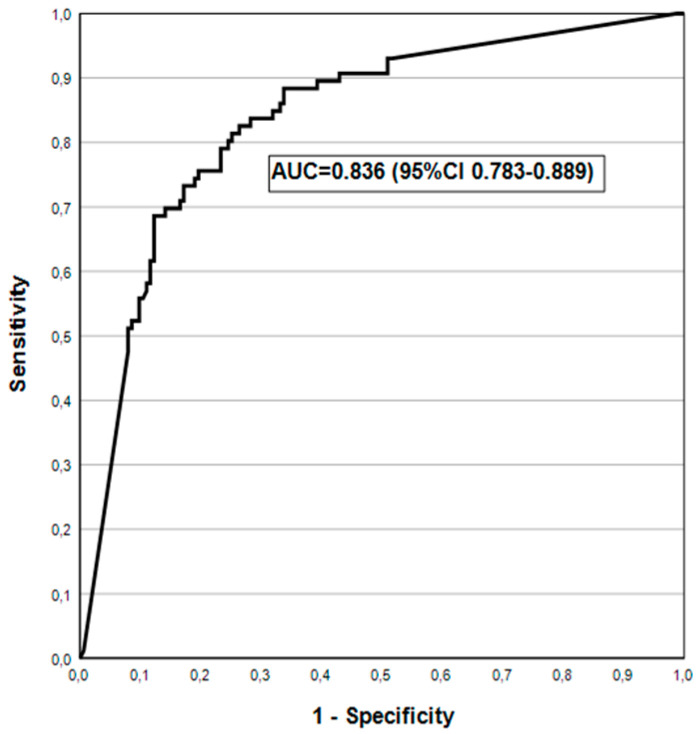
Hp IgG Ab test in detecting biopsy-confirmed Helicobacter pylori in ROC analysis.

ROC analysis for Helicobacter IgG Ab test in detecting biopsy-confirmed Hp in the antrum and/or corpus is illustrated in Figure 3. DA of GPQT in Hp-detection is also very good: AUC = 0.836 (95% CI 0.783–0.889).

**Figure 4:**
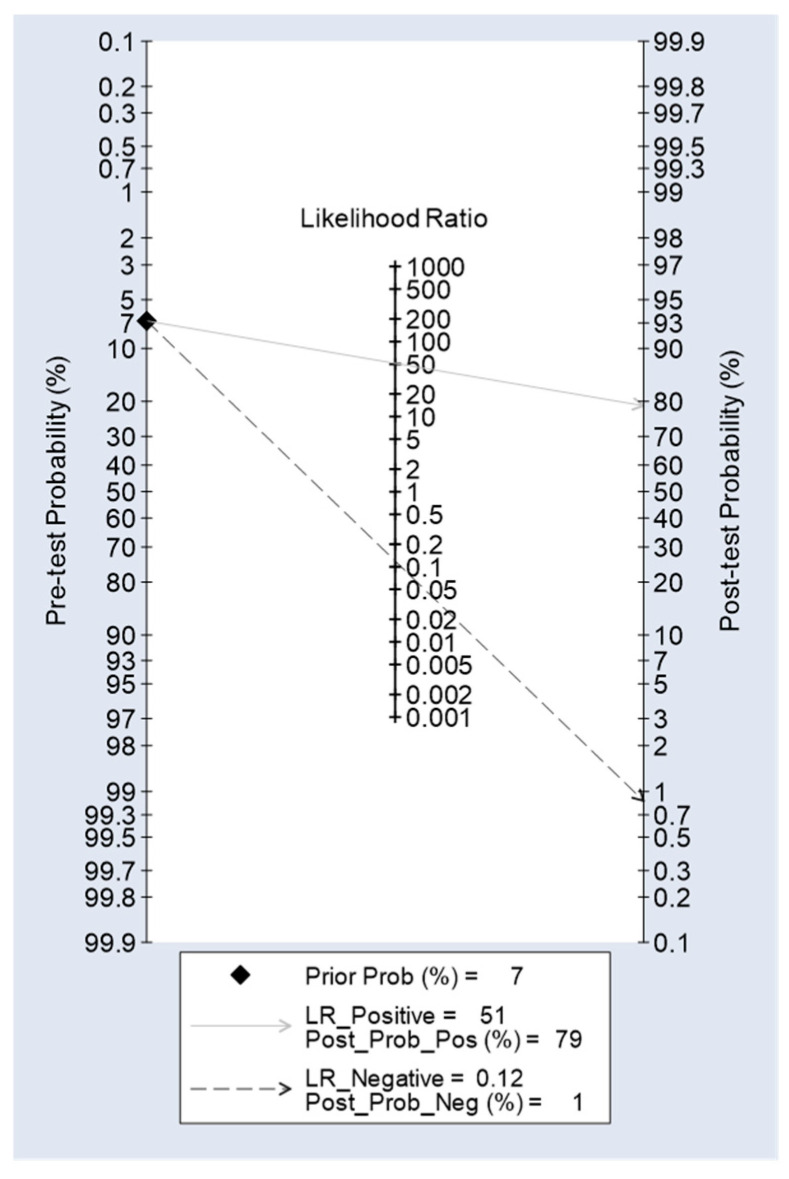
Fagan’s nomogram for GastroPanel^®^ Quick test as predictor of AGC in a population.

The Fagan’s nomogram illustrated in Figure 4 was drawn by entering the diagnostic indicators of PGI for the AGC endpoint (Table 6) [38]: (i) the pre-test probability, 0.068; (ii) LR+, 51.2, and (iii) LR-, 0.12. The post-test probabilities in the Fagan’s nomogram indicate that AGC diagnosis of the GPQT predicts AGC in a comparable population with the likelihood of 79%, whereas this likelihood is <1%, if the GPQT result is negative for AGC.

## 4. Discussion

The present trial is the first clinical validation study of the GastroPanel^®^ Quick test (GPQT) [35,36]. The results clearly confirm that GPQT favorably competes in DA with the previous GP ELISA-versions in large-scale clinical and screening settings worldwide [16,17,18,19,20,21,22,23,24,25,26,27,28,29]. In the most recent meta-analysis (in 2022) including 49 studies and 22,597 patients, GP test had pooled SE of 0.70 (95% CI = 0.64–0.76) and pooled SP of 0.93 (95% CI = 0.90–0.95), with AUC = 0.900 (95% CI = 0.170–1.000) in diagnosis of AGC [32]. Meta-regression disclosed publication year (<2008>) as the only significant study-level covariate of heterogeneity, implicating that the GP test performance in the studies published after 2008 e.g., [25,26,27,28,29,40,41,42,43] is constantly improving e.g., [18,19,20,21,22,23,24]. For unified-GP, AUC values exceeding 0.950 have been reported [26,33]. This posed substantial challenges for the designers of the GPQT [35,36], which should demonstrate that DA is not inferior to the latest ELISA test version [26,27,33,41].

Several good reasons exist, however, to justify complementing the GP-ELISA test [15,16] with a POC-test format [35,36]. While capable of offering the same range of diagnoses as the GP-ELISA test but at a single clinical appointment, the GPQT test offers several advantages that all have one feature in common: substantial savings in health care costs. These issues are increasingly important in countries with a heavy burden of GC [1,2,9,11]. These arguments were relevant enough to convince Fortis Hospital in India to approve the novel GPQT for clinical testing in their gastroenterology clinic.

A cohort of 266 gastroscopy referral patients consented to participate in this study (Table 1). As compared with a similarly designed study in Finland (for unified-GP) [33], these two cohorts share similarities (mean age, Hp-eradication history), but also important differences, particularly in the usage of PPI-medication (42% vs. 74%) and frequency of symptoms due to high acidity (33.1% vs. 97.4%). These differences make us anticipate differences also in the GP test results [33]. Such a large difference between the two cohorts was detected in their prevalence of Hp-infection: 6.7% in Finland (33) and 39.8% in India (Table 2). However, even the latter is not among the highest encountered in GP studies, e.g., in Kazakhstan [42] and in Russia [29,41], both showing *Hp*-prevalence close to 70%. One might expect that testing populations with such a divergent risk profile [16] would make the GP testing perform differently [29,33,41,42]. However, this does not seem to be the case, as confirmed by the three meta-analyses [30,31,32], all failing to demonstrate the geographic study origin as a significant source of heterogeneity. This is in perfect agreement with the results of the present study where the DA of the GPQT was practically identical with the DA of unified-GP test [33], despite these dissimilarities in the study cohorts. This is another argument in favor of the uniform performance of the GP test, which does not depend on the target populations [12,16,18,19,20,21,22,23,24,25,26,27,28,29,33,40,41,42,43].

The biomarker levels in the five diagnostic GPQT profiles disclosed some irregularities (Table 2) that deserve a mention here [26,27,33,41]. Referring to the manufacturer recommended reference values [15,16,27,35], of note are slightly increased levels of PGI that exceed the upper cut-off (160 μg/L) in the “normal” and “Hp-gastritis” categories, and the levels of G-17b that are clearly higher than expected (normal range: 1–7 pmol/L), in these two categories [15,35]. This increase is explained by two well-known features of the GP biomarkers [15,16,35,44]. PGI and G-17 are biomarkers of inflammation that increase in ongoing Hp-infection and return to normal after successful *Hp*-eradication [15,16,44]. Also, PPI-medication causes a substantial increase of both PGI and G-17 [15,16,44]. Almost 75% of the patients in our cohort used PPI-medication, which would neatly explain the elevated PGI and G-17b levels in an otherwise normal GPQT profile (Table 2). These same deviations are seen when the biomarker levels are stratified by the 5 diagnostic USS categories in the biopsies (Table 3). Discontinuation of the PPI medication 7 days before GP testing would result in the disappearance of this irregular (G-17 and PGI) biomarker profile [16,33,44].

The role of G-17 as an integral component of the GP test has been discussed in detail in two reviews [12,16]. Importantly, G-17 biomarker plays a dual role: (i) it measures the activity and mucosal integrity of the antrum directly, and (ii) functional activity and mucosal structure of the corpus indirectly [16]. Accordingly, the use of G-17 as a stand-alone marker cannot separate between (a) AGA, (b) AGP, and (c) level of gastric acid output [16]. The latter includes disturbances in acid output either through the use of PPI medication or due to mucosal atrophy in cases of AGC and AGP [12]. To make accurate distinction between these conditions, the full 4-biomarker GastroPanel^®^ test is needed, including biomarkers that measure both topographic stomach sites (antrum and corpus) directly and not only indirectly, i.e., G-17, and PGI & PGII, respectively [16]. For the diagnosis of AGA by G-17, the reader is referred to the discussion of Table 5 results that follows. A brief account is given here on the biomarker profiles caused by PPI medication, because pertinent to the present cohort with almost 75% of PPI-user frequency (Table 2).

Any gastric acid-suppressive medication will inevitably interfere with the profile of the GP biomarkers [16]. PPI and H_2_-blockers effectively reduce gastric acid production by parietal cells [12,16,27,44]. This increases the output of G-17b and that of PGI and PGII. Once the PPI/H_2_-blocker treatment is discontinued, it takes 4–10 days for acid output and G-17b levels to normalize. However, PGs respond more slowly, and PGI and PGII levels may remain above the cut-off values for up to 2–3 weeks [16,44]. An abrupt termination of long-term PPI medication is typically followed by rebound acid hypersecretion, frequently accompanied by symptoms of hyper-acidity and extremely low levels of G-17b [16,44]. To ensure an unbiased assessment of the biomarker profiles, the manufacturer recommends that the patient discontinues any acid-suppressive treatment for 10 days before serum/plasma sampling [16,27,44]. This withdrawal of PPI or H_2_-blocker medication is not always possible because of intractable clinical symptoms of acidity, and to acknowledge this, the latest version of GastroSoft^®^ was designed to consider the continued use of PPI medication [16,44]. In this context, essential information includes (i) an accurate recording of the PPI/H_2_-medication, (ii) whether the medication was discontinued, and if so, (iii) for how many days before GP sampling. In the present cohort, the later information was incomplete, unfortunately (Table 2).

The two most widely used histological classifications of gastritis are: (1) the Updated Sydney System (USS) [9,11] and (2) the OLGA/OLGIM staging [45]. The GP test was optimized for use with the USS, both including 5 diagnostic categories [9,11,15,16,26,33,35], which makes it straightforward to calculate the agreement between the two methods. Despite the good inter-rater reproducibility of the USS [9,11,16], challenges are encountered in classifying mild atrophy even by experienced pathologists [9,11,16]. In this study, the original (Fortis) and revised (Finland) USS classification had a very good agreement: weighted kappa, κ_w_ = 0.844 (95% CI 0.800–0.878), and OA = 83.9% (95% CI 79.3–88.4%), formally ranking as an “almost perfect agreement” [46]. Divergent ranking of the AGA in Fortis and in Finland; 49 and 21 cases, respectively, caused most of the observed disagreement.

GPQT and USS classification were closely agreed: κ_w_ = 0.823 and OA of 71.4% (95% CI 65.4–77.0%) (Table 4), representing “almost perfect” (0.8–1.0) and “substantial” (0.6–0.8) agreement, respectively [46]. This lends further support to the statement that the GP test bears a close concordance with the USS classification [12,15,16,17,18,19,20,21,22,23,24,25,26,27,30,31,32,33,47]. In this context, a word of caution must be stated against the use of OLGA staging [45] for GP validation. Because OLGA staging combines different grades of AGA and AGC into one and the same OLGA stage [45], this obscures an accurate linking of specified GP biomarker profiles to individual OLGA stages [15,16,30,32,47].

GastroSoft^®^ application defines biomarker profiles distinct for AGA (low G-17b and G-17s, Hp+) and AGC (low PGI, PGI/PGII ratio, high G-17b) [15,16,26,27,33,44,47], which can be used for testing the DA of GPQT separately for biopsy-confirmed AGA and AGC (Table 5). The SE of the AGA profile in diagnosis of AGA (n = 21) is low (14.3–30.0%), whereas SP is high (>99%), resulting in AUC values between 0.569 and 0.648. This is in sharp contrast to AGC, where GastroSoft^®^ AGC profile detects the biopsy-confirmed AGC2+ with 100% SE and 95.4% SP, equivalent to AUC = 0.977. The DA for AGC (all grades) is only slightly inferior (AUC = 0.933). The reasons for the divergent DA for AGA and AGC are explained by the inherent characteristics of the GastroPanel^®^ biomarkers, as explained in the following.

First, the GP test has been designed to include biomarkers that measure the function and structure of both the antrum (G-17b, G-17s) and the corpus (PGI, PGII, PGI/PGII ratio) [12,15,16,33,44,47]. Completely different GP biomarker profiles reflect this topographic location of AG, and because of this, AGA and AGC should not be combined as a single entity “chronic atrophic gastritis” while assessing the DA of the GP test [12,15,16,33,44,47]. Second: As also shown in the present study, mild AGA and mild AGC are poorly reproducible histopathological diagnoses [9,11,16,44,47] and should never be used as the endpoint in these DA calculations [16,47]. Instead, only moderate/severe AG (AGC2+, AGA2+) should be used.

Third: Low G-17b values are due to high acid output of the corpus in most patients, while AGA is a far more uncommon cause of low G-17b [9,11,15,16,19,25,26,27,33,44,47]. In the present cohort, there were 21 cases classified as AGA (Table 4), whereas 259 patients reported symptoms of high gastric acid output (Table 1). Because of this dual regulation of the antral G-17, the fasting level (G-17b) of this biomarker cannot accurately indicate only AGA [12,15,16,19,33,44,47]. In this respect, protein-stimulated G-17 (G-17s) is more helpful, but truly low levels of G-17s are encountered only in moderate/severe AGA when the antral G-cells are about to disappear [30,47]. Due to these inherent physiological characteristics of the GP biomarkers, the DA of the test in detection of AGA never reaches the high level achieved in diagnosis of AGC [16,25,26,27,33,44,47].

When the cut-off values of the biomarkers were used instead of the GastroSoft^®^ AGA/AGC profiles, the differences in the DA for AGA and AGC persist (Table 6). G-17b cut-off (1.0 pmol/L) has very low SE (9.5%) but very high SP (>96%) in diagnosing AGA (any grade), which improve to 20% and 97.1%, respectively, for the AGA2+ endpoint. Of the two cut-offs tested for PGI (15 and 30 μg/L), the latter is clearly superior (by >20%) in SE for both AGC (all) and AGC2+, while SP is unaffected. For the AGC2+ endpoint, the highest DA (AUC = 0.983) was obtained with PGI at 30 μg/L cut-off, instead of the PGI/PGII ratio (3.0 cut-off), which proved to be the best in our recent study [33]. These outstanding AUC values are confirmed also by ROC analysis for PGI and PGI/PGII (Figure 1 and Figure 2), both being clearly superior to the pooled AUC values (AUC = 0.900) in the latest meta-analysis [32]. Thus, in this setting of gastroscopy referral patients in India, the new GPQT exceeds the DA of the GP testing reported in most of the previously published clinical studies [16,19,20,21,22,23,24,25,26,27,28,29,30,31,32,33,40,41,42,43].

The Hp IgG ELISA of the unified-GP test was recently tested in a population with a high (64%) prevalence of *Hp*-infection, showing a 91% agreement with *Hp*-detection in biopsies [27]. In the present cohort with *Hp*-prevalence of 26.1% (Table 3), the DA of the GPQT *Hp* IgG Ab test had AUC = 0.836 (Figure 3). Although slightly inferior to *Hp* IgG Ab ELISA testing, this AUC value is still ranked within the highest accuracy category of the ROC analysis [27]. A detailed discussion on the extensive subject of *Hp*-testing falls outside the scope of this communication.

The figures in the Fagan’s nomogram [39] implicate that an AGC result in GPQT test predicts AGC in a population with 79% likelihood, whereas this likelihood is below 1% if the GPQT result is negative for AGC (Figure 4). These positive and negative post-test probabilities are almost identical with those reported recently for the unified-GP test in Finland [33]. This is yet another indication on the uniform performance of the GP test, irrespective of the test version and the target population [16,47]. Assuming that the present cohort is representative of the local population in this region, the pre-test probability of AGC (6.8%) should closely reflect the AGC prevalence in this region of India [39].

As to the potential limitations of this study, one could consider the following as such: (i) a relatively limited size of the cohort (n = 266/249), (ii) failure to have a more in-dept focus on diagnosis of *Hp*-infections (i.e., whether current or past), (iii) failure of GPQT to diagnose GC thus leading to potential delays in diagnosis, and (iv) failure of this test to diagnose pathologies of the esophagus and duodenum. In a closer look, however, the concern about these seeming limitations proves to be unnecessary. Accordingly, (i) in GastroPanel^®^ DA studies, the cohort size did not prove to be an independent study-level covariate in formal meta-regression included in the recent meta-analyses [30,31,32], implicating that the DA of this test does not depend on cohort size.

As to the diagnosis of *Hp*-infections, the company has two distinct quick tests available (*Helicobacter pylori* quick test and BIOHIT Helicobacter pylori UFT300) for the POC detection of *Hp* in the gastric biopsies. In the present study, these were not used for two simple reasons: (i) these tests do not belong among the routine clinical practice of the hospital, and (ii) both these quick tests have a very close correlation to GPQT. Thus, the present study was designed to simulate the real-life management of the target patients as much as possible, and adding an extra biopsy in the study protocol was not felt feasible. GPQT (like the ELISA versions) is known to give adequate results in the *Hp*-status of the patient, i.e., ongoing infection, previous exposure, successful eradication, failed eradication, as confirmed in the previous studies [27,44], making additional testing by urea-based quick tests superfluous in the present study.

GastroPanel^®^ is not a diagnostic test for GC, as repeatedly emphasized in all study reports and reviews [16,29,30,31,32,40,41,42,43,44,45,46,47], but intended for diagnosis and screening of the GC risk conditions (*Hp* and AG) [16]. The rational is that early diagnosis of these conditions and the referral of the risk patients to endoscopy, will lead to early diagnosis of GC precursors and early-stage GC. Thus, instead of causing delays in GC diagnosis, a systematic GastroPanel^®^ screening of subjects at risk would enable both primary prevention and early diagnosis, as discussed in detail elsewhere [16].

Finally, the concern of missing some pathologies in the esophagus and duodenum by stand-alone GastroPanel^®^ testing is relevant, but only in part concerning disorders of the duodenum. GastroPanel^®^ is not a test for duodenal disorders, which are not the subject of this validation study, and would be diagnosed by EGD which the patients were referred to at the clinic. With regard to esophageal conditions, most importantly cancer precursors like GERD/NERD and Barrett’s esophagus, GastroPanel^®^ test also gives important information about their risk, as previously explained [16]. In brief, high acid output together with low G-17 can indicate an increased risk of GERD, and as such is regarded (by the test manufacturer) as an indication to perform EGD [16].

## 5. Conclusions

The results of this first clinical validation study on the new GastroPanel^®^ Quick test clearly confirm that the DA of the new GPQT test favorably competes with the ELISA test versions in diagnosis of AGC and *Hp*-infection in patients referred for gastroscopy [30,31,32,33,47]. As already well established for the other GP test versions [15,16,33,47], G-17b alone is not an accurate biomarker of AGA. Being closely concordant with the biopsy histology, GPQT offers an outstanding new tool for the POC diagnosis of GC risk conditions (AG and *Hp*-infection) at a single clinical appointment. Apart from its technical excellence, the adoption of GPQT in the clinical management of the patients at high-risk for GC offers several advantages: (i) obtains cost savings by reducing unnecessary clinical appointments, (ii) speeds up the access of the patients to clinical triage (EGD) when truly indicated, as well as (iii) allows more rational allocation of the endoscopy resources when unnecessary EGD examinations are avoided [35].

## Figures and Tables

**Table 1:** The clinical information requested in the GastroPanel^®^ Quick test referral form.

**Characteristics**	**Number of Patients** **(n = 266)**	**Per Cent of Total** **100%**
Gender:		
Women	135	50.8
Men	131	49.2
Age (Median; Range)	52.0 years	(18–92 years)
History recorded in GastroPanel^®^ (GP) referral form:		
1. Helicobacter diagnosed:		
Hp diagnosed in GP testing	15	5.6
Hp diagnosed withing one year	25	9.4
Hp never diagnosed	223	83.8
Responder does not know	3	1.2
2. Helicobacter eradication, if done:		
Yes, eradication successful	12	38.7
Eradication not successful	12	38.7
Responder does not know	7	22.6
3. Use of PPI-medication:		
No PPI-medication	68	25.6
Continuous use of PPI-medication	197	74.1
Responder does not recall	1	0.3
4. Symptoms of high acidity (heartburn)		
No symptoms of high acidity	6	2.3
Continuous symptoms of high acidity	259	97.4
Data missing	1	0.3

**Table 2:** Biomarker levels stratified by the categories of GastroPanel^®^ Quick test.

GPQT Diagnosis	No. of Cases	PGI (M ± SD)	PGII (M ± SD)	PGI/PGII (M ± SD)	G-17b (M ± SD)	HpAb (M ± SD)
Normal	136	165.6 (80.2)	17.1 (12.0)	12.3 (6.5)	23.2 (21.9)	10.8 (14.7)
Hp-gastritis	106	175.8 (79.8)	24.6 (16.5)	9.5 (6.9)	25.4 (21.1)	119.8 (51.3)
AGA	2	160.0 (22.6)	20.4 (2.9)	7.8 (0.3)	2.3 (1.2)	134.5 (40.3)
AGC	19	20.1 (9.7)	14.4 (9.7)	1.8 (1.0)	50.6 (13.2)	16.4 (35.7)
AGP	2	30.9 (6.9)	6.5 (5.0)	6.1 (3.6)	0.9 (0.0)	10.1 (5.8)
Total Series	265 *	158.2 (86.4)	19.9 (14.3)	10.4 (6.9)	25.8 (22.1)	55.7 (64.1)

AGA, atrophic antrum gastritis; AGC, atrophic corpus gastritis; AGP, atrophic pan-gastritis; * from one patient, GPQT results are not available.

**Table 3:** Biomarker levels across the five categories of the USS classification.

USS Grade	No of Cases	PGI (M ± SD)	PGII (M ± SD)	PGI/PGII (M ± SD)	G-17b (M ± SD)	HpAb (M ± SD)
Normal	146	170.5 (82.9)	18.2 (12.7)	12.1 (7.1)	23.3 (21.8)	30.0 (45.6)
Hp-gastritis	65	158.7 (78.5)	21.9 (15.6)	9.6 (6.5)	23.0 (20.5)	106.9 (62.2)
AGA	21	180.1 (88.0)	27.0 (16.5)	8.0 (4.9)	25.3 (22.3)	103.0 (69.5)
AGC	15	28.0 (40.9)	14.0 (10.8)	2.2 (2.0)	53.0 (11.6)	28.7 (54.7)
AGP	2	90.5 (77.2)	23.8 (19.4)	3.7 (0.2)	28.5 (38.9)	19.2 (18.7)
Total Series	249 *	159.0 (86.9)	19.7 (14.0)	10.4 (7.0)	25.3 (22.1)	56.0 (63.9)

USS, updated Sydney system; AGA, atrophic antrum gastritis; AGC, atrophic corpus gastritis; AGP, atrophic pan-gastritis; * Gastroscopic biopsies are not available from 17 patients.

**Table 4:** Agreement between GastroPanel^®^ Quick test and the USS classification.

GPQT Diagnosis	The Updated Sydney System (USS)					
	Normal	Hp-gastritis	AGA	AGC	AGP	Total
Normal	109	13	4	0	1	127
Hp-gastritis	35	52	13	1	0	101
AGA	0	0	2	0	0	2
AGC	2	0	1	14	0	17
AGP	0	0	1	0	1	2
Total	146	65	21	15	2	249
	Overall agreement (OA): 178/249; 0.714 (95% CI 0.654–0.770);					
	* Weighted kappa (κ_w_): ICC = 0.823 (95% CI 0.773–0.862);					

* Weighted kappa (ICC with following set-up: parallel model, two-way random effects, consistency).

**Table 5:** Diagnostic accuracy of GastroSoft AGA and AGC profiles^@^ in diagnosis of biopsy-confirmed AGA and AGC.

GPQT Profile/ USS Endpoint	No of Cases (USS)	Sensitivity	Specificity	PPV	NPV	AUC
^@^ AGA Profile:						
* AGA	21	14.3 (3.0–36.3)	99.6 (97.6–100.0)	75.0 (19.4–99.4)	92.7 (88.6–95.6)	0.569 (0.492–0.646)
AGA2+	10	30.0 (6.6–65.2)	99.6 (97.7–100.0)	75.0 (19.4–99.4)	97.1 (94.2–98.8)	0.648 (0.498–0.798)
^@^ AGC Profile:						
** AGC	17	88.2 (63.6–98.5)	98.3 (95.6–99.5)	78.9 (54.4–93.9)	99.1 (96.9–99.9)	0.933 (0.853–1.000)
AGC2+	8	100 (63.1–100)	95.4 (92.0–97.7)	42.1 (20.3–66.5)	100 (98.4–100)	0.977 (0.964–0.990)

^@^ Diagnosis made by the GastroSoft^®^ application as AGA or AGC; * Biopsy: AGA of any grade; AGA2+ (moderate/severe AGA); ** Biopsy: AGC of any grade (2 AGP included); AGC2+ (moderate/severe AGC).

**Table 6:** Performance indicators of G-17b (1.0 pmol/L cut-off), PGI (15 and 30 μg/L cut-off) and PGI/PGII (3.0 cut-off) in diagnosis of biopsy-confirmed AGA and AGC.

Endpoint	Sensitivity	Specificity	PPV	NPV	AUC
G-17b:					
* AGA	9.5 (1.1–30.4)	96.9 (93.8–98.8)	22.2 (2.8–60.0)	92.1 (87.9–95.2)	0.532 (0.467–0.598)
AGA2+	20.0 (2.5–55.6)	97.1 (94.1–98.8)	22.2 (2.8–60.0)	96.7 (93.5–98.6)	0.585 (0.454–0.716)
PGI (15):					
** AGC	52.9 (27.8–77.0)	99.1 (96.9–99.9)	81.8 (48.2–97.7)	96.6 (93.5–98.5)	0.760 (0.638–0.883)
AGC2+	87.5 (47.3–97.7)	98.3 (95.8–99.5)	63.6 (30.8–89.1)	99.6 (97.7–100)	0.929 (0.806–1.000)
PGI (30):					
** AGC	76.5 (50.1–93.2)	98.7 (96.3–99.7)	81.3 (54.4–96.0)	98.3 (95.7–99.5)	0.876 (0.772–0.980)
AGC2+	100 (63.1–100)	96.7 (93.6–98.6)	50.0 (24.7–75.3)	100 (98.4–100)	0.983 (0.972–0.995)
PGI/PGII:					
** AGC	64.7 (38.3–85.8)	97.4 (94.5–99.0)	64.7 (38.3–85.8)	97.4 (94.5–99.0)	0.811 (0.693–0.928)
AGC2+	87.5 (47.3–99.7)	95.9 (92.5–98.0)	41.2 (18.4–67.1)	99.6 (97.6–100)	0.917 (0.794–1.000)

* AGA of any grade; AGA2+ (moderate/severe AGA); ** AGC of any grade; AGC2+ (moderate/severe AGC).

## Data Availability

The original data including all recorded information of the study subjects and their examinations (including the results of the GPQT) are available from the study sponsor upon reasonable request.

## Abbreviations

The following abbreviations are used in this manuscript:

AG	Atrophic gastritis
AGA	Atrophic antrum gastritis
AGA2+	Moderate/Severe atrophic antrum gastritis
AGC	Atrophic corpus gastritis
AGC2+	Moderate/Severe atrophic corpus gastritis
AGP	Atrophic pan-gastritis
AUC	Area under ROC curve
DA	Diagnostic accuracy
EGD	Esophago-gastro-duodenoscopy
ELISA	Enzyme-linked immunosorbent assay
G-17	Gastrin-17; G-17b (basal), G-17s (stimulated)
GC	Gastric cancer
G-cells	Gastrin-secreting cells
GERD	Gastro-esophageal reflux disease
GP	GastroPanel^®^
GPQT	GastroPanel^®^ Quick test
H_2_	Histamin-2 receptor
*Hp*	*Helicobacter pylori*
IARC	International Agency of Research on Cancer
ICC	Intra-class correlation coefficient
K-T	Kimura-Takemoto classification
κ_w_	Weighted kappa test
LR-	Negative likelihood ratio
LR+	Positive likelihood ratio
NERD	Non-erosive reflux disease
NPV	Negative predictive values
NSAID	Non-steroidal anti-inflammatory drugs
OA	Overall agreement
OLGA	Operative link to gastric atrophy
OLGIM	Operative link to gastric intestinal metaplasia
PGI	Pepsinogen I
PGII	Pepsinogen II
POC	Point-of-care
PPI	Proton pump inhibitor
PPV	Positive predictive value
ROC	Receiver operating characteristics
SE	Sensitivity
SP	Specificity
USS	Updated Sydney System
